# Blood small extracellular vesicles derived miRNAs to differentiate pancreatic ductal adenocarcinoma from chronic pancreatitis

**DOI:** 10.1002/ctm2.520

**Published:** 2021-09-10

**Authors:** Shiwei Guo, Hao Qin, Ke Liu, Huan Wang, Sijia Bai, Shiyi Liu, Zhuo Shao, Yanan Zhang, Bin Song, Xiaoya Xu, Jing Shen, Peng Zeng, Xiaohan Shi, Hao Chen, Suizhi Gao, Jiajia Xu, Yaqi Pan, Lei Xiong, Fugen Li, Dadong Zhang, Xiaodong Jiao, Gang Jin

**Affiliations:** ^1^ Department of Hepatobiliary Pancreatic Surgery Changhai Hospital Naval Medical University Shanghai China; ^2^ 3D Medicines Inc. Shanghai China; ^3^ Department of Medical Oncology Changzheng Hospital Naval Medical University Shanghai China

**Keywords:** chronic pancreatitis, differential diagnosis, pancreatic ductal adenocarcinoma, small extracellular vesicles miRNAs

## Abstract

**Background:**

The differential diagnosis of pancreatic ductal adenocarcinoma (PDAC) from chronic pancreatitis (CP) is clinically challenging due to a lack of minimally invasive diagnosis methods. MicroRNAs (miRNAs) derived from small extracellular vesicles (EVs) in the blood have been reported as a promising diagnosis biomarker for various types of cancer. However, blood small EV miRNA signatures and their diagnostic value to differentiate between PDAC and CP remain to be determined.

**Methods:**

In this study, 107 patients with PDAC or CP were recruited, and 90 patients were finally enrolled for a training cohort (*n* = 48) and test cohort (*n* = 42). Small RNA sequencing was used to assess the expression of blood small EV miRNAs in these patients.

**Results:**

The linear model from the differentially expressed blood small EV miR‐95‐3p divided by miR‐26b‐5p showed an average sensitivity of 84.1% and an average specificity of 96.6% to identify PDAC from CP in the training cohort and the test cohort, respectively. When the model was combined with serum carbohydrate antigen 19‐9 (CA19‐9), the average sensitivity increased to 96.5%, and the average specificity remained at 96.4% of both cohorts, which demonstrated the best performance of all the published biomarkers for distinguishing between PDAC and CP. The causal analysis performed using the Bayesian network demonstrated that miR‐95‐3p was associated with a “consequence” of “cancer” and miR‐26b‐5p as a “cause” of “pancreatitis.” A subgroup analysis revealed that blood small EV miR‐335‐5p/miR‐340‐5p could predict metastases in both cohorts and was associated with an overall survival (*p =* 0.020).

**Conclusions:**

This study indicated that blood small EV miR‐95‐3p/miR‐26b‐5p and its combination with serum levels of CA19‐9 could separate PDAC from CP, and miR‐335‐5p/miR‐340‐5p was identified to associate with PDAC metastasis and poor prognosis. These results suggested the potentiality of blood small EV miRNAs as differential diagnosis and metastases biomarkers of PDAC.

## Graphical abstract

The challenge of separating pancreatic ductal adenocarcinoma (PDAC) from chronic pancreatitis (CP) is the lack of minimally invasive clinically diagnostic methods.

Blood small EV miR‐95‐3p/miR‐26b‐5p and its combination with CA19‐9 serum levels were discovered and validated to separate PDAC from CP.

These results suggested the potentiality of blood small EV miRNAs as differential diagnosis biomarkers of PDAC.

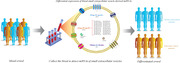

**Abbreviations**
AUC: area under the curve
CA19‐9: carbohydrate antigen 19‐9
CP: chronic pancreatitis
CT: computed tomography
EM: electron microscopy
EUS‐FNA: endoscopic ultrasound‐guided fine‐needle aspiration biopsy
EVs: extracellular vesicles
miRNA: microRNA
NTA: nanoparticle tracking analysis
OS: overall survival
PC: principle component
PCA: principle component analysis
PDAC: pancreatic ductal adenocarcinoma
qRT‐PCR: quantitative reverse transcription polymerase chain reaction
ROC: receiver operating characteristic
TCGA: The Cancer Genome Atlas
WB: western blotting

## 1 BACKGROUND

Pancreatic ductaladenocarcinoma (PDAC) is the fourth leading cause of cancer‐related death in the United States.^1^ The incidence of chronic pancreatitis (CP) is between 4 and 23/100,000,^2^ and recurrent CP may be a risk factor for PDAC.^3^ The differential diagnosis between PDAC and CP is quite frequent and challenging in the early diagnosis of pancreatic cancer, which is a major cause of misdiagnosis and mistreatment.^4^ Thus, distinguishing between PDAC and CP is an urgent need and of clinical importance to improve patient outcomes.

Currently, commonly used imaging examinations, including high‐resolution computed tomography (CT), magnetic resonance imaging, and positron emission tomography, have difficulties in distinguishing PDAC from mass pancreatitis disease.^5^, ^6^ Endoscopic ultrasound‐guided fine‐needle aspiration biopsy (EUS‐FNA) is an important invasive method for PDAC diagnosis. However, EUS‐FNA carries the risk of infection, hemorrhage, and tumor seeding. More importantly, it has a considerable false‐negative rate.^7^ Carbohydrate antigen 19‐9 (CA19‐9) is currently the widely used blood‐based tumor marker for the management of pancreatic cancer. CA19‐9 has been reported to distinguish between pancreatic cancer and benign pancreatic disease with a relatively limited sensitivity and specificity (sensitivity of 78.2% and specificity of 82.8%),^8^, ^9^ rendering it ineffective as a differential diagnosis biomarker. Thus, minimally invasive diagnosis methods and effective risk stratification approaches for the distinction between PDAC and CP need to be developed.

Extracellular vesicles (EVs) consist of nucleotides, proteins, lipids, and other molecules that are present in nearly all biological fluids, including the blood circulation.^10^, ^11^ Circulating EVs are identified as a stable source of microRNAs (miRNAs) derived from their parental cells that include cancer cells and normal cells.^12^, ^13^, ^14^ Recently, circulating small EV‐derived miRNAs have gained attention as a promising blood‐based biomarker for cancer detection.^15^, ^16^, ^17^ In pancreatic cancer, several studies have also revealed that blood small EV miRNAs could serve as potential diagnostic biomarkers.^18^, ^19^, ^20^, ^21^ In addition, Xu et al. found that plasma small EV miR‐196a and miR‐1246 levels were significantly elevated in pancreatic cancer patients as compared to healthy subjects.^22^ Moreover, Lai et al. confirmed that some small EV miRNAs (e.g., miR‐10b, miR‐21, miR‐30c, miR‐181a, and miR‐let7a) were differentially expressed among PDAC patients, normal controls, and patients with CP.^23^ However, those studies were limited by the small number of detected small EV miRNAs in blood, small sample sizes, or lack of independent validation.

In this study, the miRNA profile of blood small EVs derived from PDAC and CP patients is characterized using small RNA sequencing, and a two‐phase method (training phase and test phase) is used to discover and validate the diagnostic‐associated blood small EV miRNAs for distinguishing between PDAC and CP. In addition, a subgroup analysis regarding the association between metastasis‐associated blood small EV miRNAs and patient outcomes after surgery is conducted. The primary objective of this study is to identify blood small EV miRNAs as a minimally invasive method to differentiate between PDAC and CP.

## 2 METHODS

### 2.1 Patient enrollment and sample collection

A total of 107 patients were recruited for the training phase and test phase from June 2017 to June 2018 (Additional file 1: Figure S1). In this study, PDAC was diagnosed using enhanced CT and verified using EUS‐FNA of primary or metastatic lesions.^24^ The diagnosis of CP referred to the consensus of the American Pancreatic Association.^25^ Inclusion criteria were as follows: (1) Patients had to sign a consent form for their blood specimens and clinical information to be used in this research. (2) Patients were diagnosed with PDAC. (3) Patients were diagnosed with CP. Exclusion criteria were as follows: (1) Pancreatic cancer patients undergoing radiotherapy or chemotherapy prior to blood collection. (2) Patients with pancreatic benign tumors or borderline malignant tumors. (3) Patients with pancreatic metastases (e.g., kidney cancer metastases to pancreas). (4) Patients with pancreatic serous cystadenocarcinoma or mucinous cystadenocarcinoma. (5) Patients with CP who had presented with acute pancreatitis within the past 3 months. (6) Patients with CPs who showed a malignant tendency within the follow‐up period of 6 months. (7) Blood specimens with hemolysis levels greater than 5. After the elimination of pathology specimens from patients with non‐PDAC and no operation or serious hemolysis, a total of 90 patients were enrolled, 48 in a training cohort and 42 in a test cohort. In the training cohort, six patients were in Stage I, 10 patients in Stage II, two patients in Stage III, and 12 patients in Stage IV; in the test cohort, there were six patients in Stage I, six patients in Stage II, four patients in Stage III, and 11 patients in Stage IV. The clinical characterization of these patients in the two cohorts is shown (Table 1 and Additional file 2: Table S1). All patients with PDAC in the training cohort and the validation have received standard adjuvant therapy. The overall survival (OS) was defined as the period from the start of surgery to death. Among the PDAC patients, 46 provided complete follow‐up information (Additional file 2: Table S2).

**TABLE 1:** Clinical characteristics of PDAC and CP patients in the training and test cohorts

Characteristics	Training cohort (*N* = 48)	Test cohort (*N* = 42)	*p* value
Age (years), average	56 ± 13	53 ± 14	0.290
Gender			
Male	32	26	0.638
Female	16	16	
Categories			
CP patients	18	15	0.861
PDAC patients	30	27	
Incidence site			
Pancreatic head	15	17	0.325
Pancreatic body and tail	15	10	
Stage			
I	6	6	0.669
II	10	6	
III	2	4	
IV	12	11	
Metastasis			
M0	18	16	0.955
M1	12	11	
CA19‐9 (U/ml) of PDAC patients, average	560.7 ± 496.7	600.6 ± 506.4	0.769
CEA (ng/ml) of PDAC patients, average	5.5 ± 4.0	4.6 ± 4.3	0.395

Abbreviations: CA19‐9, carbohydrate antigen 19‐9; CEA, carcinoembryonic antigen; CP, chronic pancreatitis; PDAC, pancreatic ductal adenocarcinoma.

The blood specimens of all the patients were collected in 6‐ml vacutainers with anticoagulant (REF367863; Becton Dickinson, Franklin Lakes, NJ, USA) before surgical resection. In this study, the blood specimens were collected at different periods (training cohort, June 2017–December 2017; test cohort, February 2018–June 2018). The small RNA sequencing and analysis were independently performed in the training cohort and test cohort. After identifying the potential biomarkers to separate PDAC patients from CP patients in the training cohort, the validation was carried out in the test cohort (Additional file 1: Figure S1). All of the tissue specimens were confirmed using surgical pathology. The investigational protocol was approved by the Institutional Review Board of the Changhai Hospital of Naval Medical University (CHEC2018‐039). All of the patients from the Changhai Hospital of the Naval Medical University were provided written consents to be signed for their blood specimens and clinical information to be used in this research.

### 2.2 Plasma isolation

For the plasma isolation, the collected blood specimens were centrifuged at 1600*g* for 10 min at 4°C, after which the hemolysis levels were determined and recorded. The blood specimens with hemolysis levels below 4 were used. The collected supernatant was centrifuged again at 16,000*g* for 15 min at 4°C, and then 1 ml of supernatant was transferred into a fresh 1.5‐ml tube and stored at −80°C.

### 2.3 Blood small EV isolation

As described in our previous report,^26^ the 3D Medicines isolation reagent (3DMed, Shanghai, China) was used to collect the blood small EVs. In brief, plasma samples that were stored in a refrigerator at −80°C were placed in a metal bath incubator set to 37°C for static incubation. Generally, the plasma melts after 5 min of incubation. The isolated plasma specimens were centrifuged at 12,000*g* for 10 min at 4°C after incubation. Then, the supernatant was transferred to a 0.45‐μm tube filter (Costar, Corning, NY, USA) and centrifuged at 12,000*g* for 5 min at 4°C; then, the filtrate was transferred to a 0.22‐μm tube filter (Costar) and centrifuged at 12,000*g* for 5 min at 4°C. Next, one‐quarter volume small EV isolation reagent (3DMed) was added to the supernatant in a fresh 1.5‐ml tube. The mixture was incubated for 30 min at 4°C and centrifuged at 4700*g* for 30 min at 4°C. Then, the supernatant was removed and the pellets containing total blood small EVs were resuspended in 0.2 ml phosphate‐buffered saline.

### 2.4 Characterization of blood small EVs

To analyze the characterization of the blood small EVs, the widely used methods include western blotting (WB), nanoparticle tracking analysis (NTA), and electron microscopy (EM). WB was performed as described previously,^26^ utilizing the following primary antibodies: anti‐Alix antibody (1:1000, Cell Signaling Technology, Danvers, MA, USA), anti‐cluster of differentiation 63 (CD63) (1:2000, Abcam, Cambridge, UK), anti‐TSG101 polyclonal antibody (1:500, Absin Bioscience Inc., Shanghai, China), and anti‐Calnexin antibody (1:1000, Cell Signaling Technology). In addition, NTA and EM were performed as described previously.^26^

### 2.5 Small RNA isolation from blood small EVs

The miRNAs were extracted from the blood small EVs using the miRNeasy Serum/Plasma Kit per the manufacturer's protocol (QIAGEN, Shanghai, China). The miRNA quality, yield, and distribution were analyzed using the Agilent 2100 Bioanalyzer with Small RNA Chips (Agilent, Savage, MD, USA).

### 2.6 Small RNA sequencing libraries preparation

Please refer to the previous study.^26^ The small RNA libraries were constructed using the NEBNext, Multiplex Small RNA Library Prep Set for Illumina (New England Biolabs, Ipswich, MA, USA) as per the manufacturer's protocol. A total of 20–25 libraries were pooled into a single sequencing lane and sequenced using the Illumina HiSeq PE150 analyzer.

### 2.7 Bioinformatic analysis

The adaptor sequences of the reads were trimmed using an in house developed program. Then, the trimmed reads were mapped to hg19 by BWA ^27^ 0.7.12‐r1039. The number of reads mapped to each miRNA locus annotated in miRBase ^28^ v21 was calculated. To normalize the expression across specimens, a set of housekeeping miRNAs that possessed at least one read in all of the specimens was selected. The 75th percentile of the housekeeping genes in each specimen was calculated as the size factor, similar to the method presented in DESeq2.^29^ The normalized expression was calculated according to the following equation:

Normalized expression = number of reads mapped to the miRNA/size factor.

### 2.8 Measurement of blood small EV miRNA expression using quantitative reverse transcription polymerase chain reaction

According to the manufacturer's instructions, we quantified the relative expression levels of small EV miR‐95‐3p and miR‐26b‐5p using TaqMan™ Advanced miRNA cDNA Synthesis Kit (Applied Biosystems, Foster City, CA, USA) in the clinical blood specimens of PDAC and CP patients. All the primers of miR‐95‐3p and miR‐26b‐5p were obtained from Applied Biosystems. Data were analyzed using the 2^−△△Ct^ method.

### 2.9 Statistical analysis

For principal component analysis (PCA), the normalized expression of each miRNA was scaled to avoid the bias of highly expressed miRNAs. For each miRNA, the maximum normalized expression was identified, and the value was divided by the maximum to be scaled. PCA was performed on the scaled data by prcomp function in R 3.3.3. Then, specimens were classified into PDAC and pancreatitis groups, and the log2 fold change was calculated as the mean of the normalized count of both groups. Linear models in R package limma v3.36.2 were used to analyze the differentially expressed miRNAs. False discovery rate < 0.05 and log2 fold change > 1 were set as the cutoff, and the normalized count of each miRNA was used as a predictor to separate PDAC and pancreatitis specimens. To test the performance of miRNAs, the area under the curve (AUC) was calculated using R package pROC v1.12.1. To investigate which miRNAs affect the metastasis of PDAC, the differentially expressed analysis of the miRNAs was performed on the carcinoma specimens. *p <* 0.05 was set as the cutoff, and the AUC was calculated from the normalized count.

To assess the sensitivity and specificity of the blood small EV miRNA model, a pathological diagnosis is regarded as the gold standard. In the PDAC and CP differential diagnosis: Sensitivity = True Positive/(True Positive + False Negative); Specificity = True Negative/(True Negative + False Positive).

To establish the Bayesian network, the normalized expression of each miRNA was discretized according to the median of the miRNA. The values larger than or equal to the median were classified as high, or were otherwise classified as low. Then, the Bayesian network was trained with the discretized values by the “bn.fit” function in the R package bnlearn v4.2 using the “tabu” algorithm. The experimental validated targets of the miRNAs were searched, and pathway enrichment was performed using mirPath ^30^ v3. The differences in clinical characteristics, including age, gender, CA19‐9 level, and carcinoembryonic antigen level of patients in the training and test cohorts, were assessed by the Student's *t* test. The chi‐square test was used to analyze differences among the other clinical characteristics between the two cohorts. The Kaplan–Meier plot of The Cancer Genome Atlas (TCGA) data was performed using the OncoLnc.^31^ *p <* 0.05 was considered statistically significant.

## 3 RESULTS

### 3.1 Characterization of the blood small EVs

**FIGURE 1:**
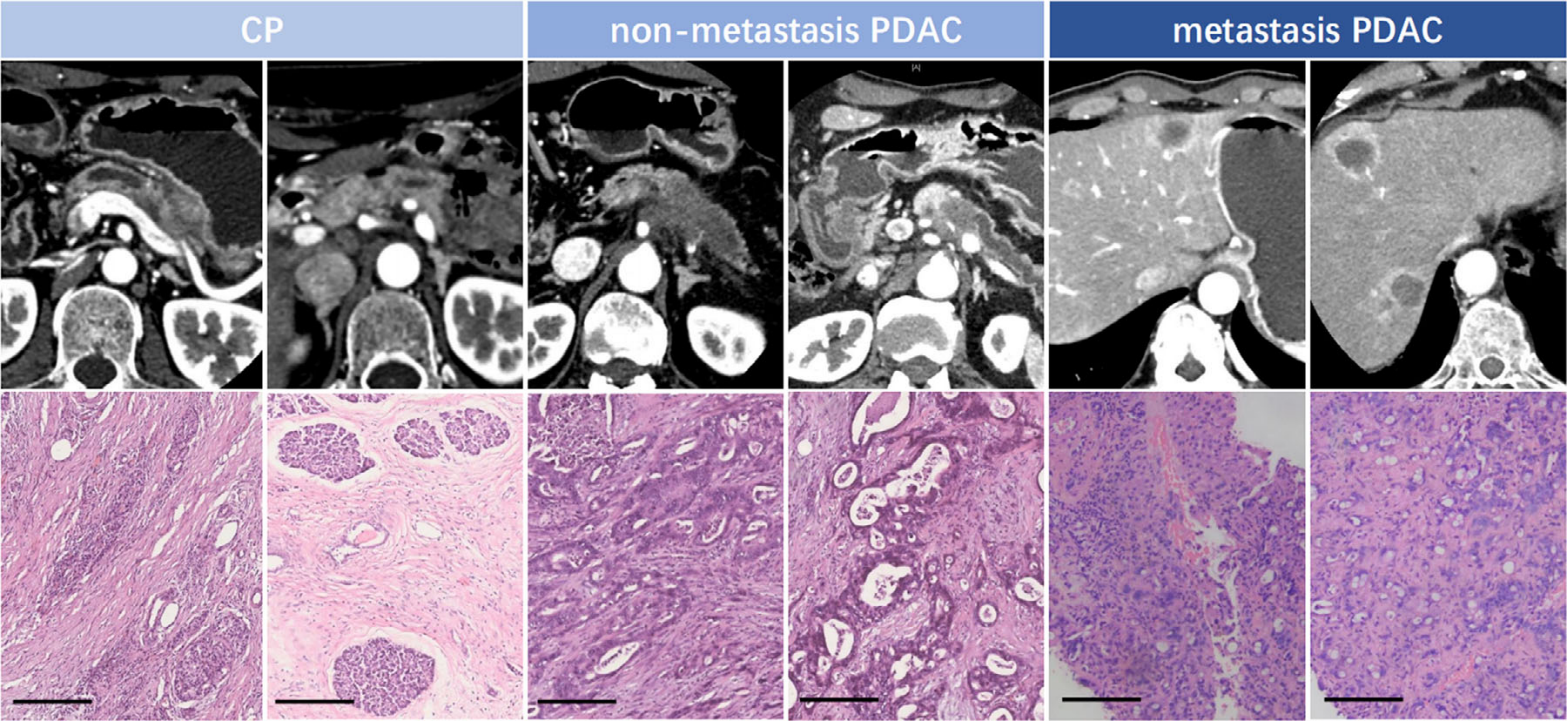
Representative imaging features and pathological information of patients with CP, nonmetastasis PDAC, and metastasis PDAC. Representative imaging results of the patients were performed using enhanced CT. Hematoxylin and eosin staining results of tumor tissue specimens derived from patients are shown. Magnification is ×200. Scale bar, 200 μm

**FIGURE 2:**
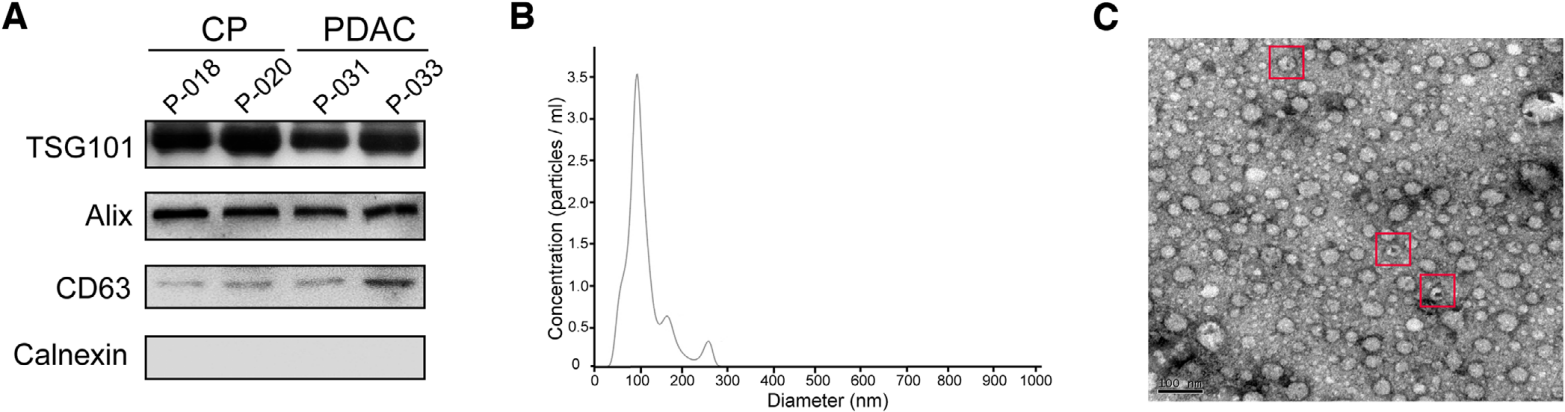
The characterization of the blood small extracellular vesicles. (A) The expression levels of the small EV common protein markers TSG101, Alix, CD63, and negative marker Calnexin in the small EVs of four representative specimens were assessed using western blotting. (B) Nanoparticle tracking analysis result from a representative small EV specimen is shown. (C) The blood small EVs image from a representative specimen was taken using an electron microscopic analysis. The representative small EV morphologies are highlighted with the red boxes

In this study, a total of 90 subjects with PDAC or CP were enrolled into the training cohort (*n* = 48) and test cohort (*n* = 42). The representative imaging features and pathological information of the patients are shown in Figure 1. After collecting the plasma from the blood specimens of the patients, the small EVs were successfully isolated. Following the guideline from the International Society for Extracellular Vesicles, WB, NTA, and EM were conducted to characterize the blood small EVs. According to the Minimal Information for Studies of Extracellular Vesicles 2018,^32^ three small EV protein markers, including Alix, TSG101, CD63, and negative marker Calnexin were evaluated in the four representative blood small EV specimens using WB. As expected, the common markers Alix, TSG101, and CD63 were present in all representative blood small EV specimens, but not Calnexin (WB; Figure 2A). The size distribution of the blood small EVs was assessed using the NTA analysis and displayed a peak around ∼100 nm (NTA; Figure 2B). In addition, EM analysis of the representative specimen showed that blood small EVs isolated in this study were oval or bowl‐shaped (EM; Figure 2C). This result was consistent with past observations of the exosomal morphologies.^33^

### 3.2 Profiles of blood small EV miRNAs were distinct among patients with CP and PDAC

**FIGURE 3:**
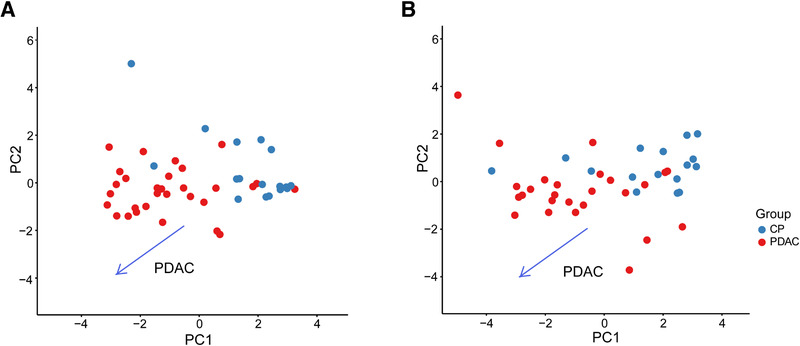
Distinct blood small EV miRNA profiles between CP patients and PDAC patients. (A) The PCA showed the blood small EV miRNA profiles on PC1 and PC2 in the training cohort. (B) The PCA showed the blood small EV miRNA profiles on PC1 and PC2 in the test cohort. Population groups (PDAC patients and CP patients) are denoted by color

To explore the potential dominant factors that influenced the blood small EV miRNA expression profile among the patients with CP or PDAC, PCA was performed on the normalized expression of all the specimens in the training cohort (Figure 3A). In the two‐dimensional space constructed by principal component 1 (PC1) and PC2, the two major principal components accounted for the largest variation among the blood small EV miRNA profiles. The PDAC patients were generally allocated to the bottom left corner region with a clear boundary of separation from the CP patients, which were distributed on the upper right region (Figure 3A). Similar distributions of PCA were also observed in all specimens of the test cohort (Figure 3B). The PCA revealed the huge dissimilarity in the blood small EV miRNA profiles between the CP and PDAC patients, showing the plausibility of identifying small EV miRNA markers to distinguish between the two patient types.

In addition, blood specimens from 12 healthy participants were collected in addition to the test cohort. To explore the discrimination capability, healthy participants were merged with the PDAC and CP specimens from the test cohort. The PCA result showed that the healthy controls could be separated from the PDAC and CP specimens (Additional file 1: Figure S2).

The profiles of blood small EV miRNAs detected using small RNA sequencing in the training cohort and test cohort are shown in two heatmaps (Additional file 1: Figure S3).

### 3.3 Ratio of miR‐95‐3p and miR‐26b‐5p from the blood small EVs was identified to distinguish PDAC from CP

**FIGURE 4:**
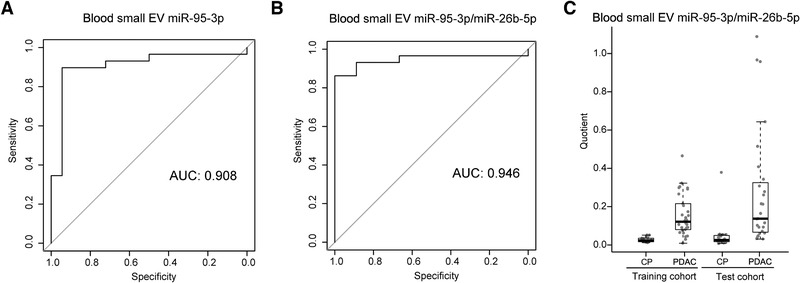
Effects of the blood small EV miR‐95‐3p/miR‐26b‐5p to distinguish between PDAC and CP patients. (A) The AUC of the ROC curves of the blood small EV miR‐95‐3p for distinguishing between PDAC and CP patients in the training cohort. (B) The AUC of the ROC curve of the blood small EV miR‐95‐3p/miR‐26b‐5p for distinguishing between PDAC and CP patients in the training cohort. (C) The distribution of levels are shown as boxplots for the quotient of the blood small EV miR‐95‐3p over miR‐26b‐5p

To identify potentially differential diagnostic biomarkers between patients with PDAC and CP, the prediction accuracy of each blood small EV miRNA was performed using the normalized expression with several filter conditions (miRNA expressed in all specimens; mean value of miRNA normalized expression > = 50; log2 fold change > 1; *p <* 0.05), and was measured using the AUC of the receiver operating characteristic (ROC) curve. The results of the top 15 candidate blood small EV miRNAs sorted by the AUC are shown for the training cohort (Additional file 2: Table S3), where miR‐95‐3p had the highest AUC at 0.908 (Figure 4A). Because the small EV miRNA in blood may be derived from various origins, using the same normalization size factor for all of the miRNAs might not properly normalize each individual miRNA. Similar to the normalization concept of quantitative polymerase chain reaction (qPCR), a pair of miRNAs can be selected in which one miRNA is considered the target gene and the other is considered the reference gene. The quotient calculated by dividing the target gene expression by the reference gene expression can be considered a joint marker. We selected highly and differentially expressed miRNAs (mean expression ≥ 50 between PDAC and CP; Additional file 2: Table S4), and calculated the prediction AUC of miR‐95‐3p divided by all of the possible pairs of these selected miRNAs in the training cohort (Additional file 2: Table S5). Among all of the pairs of the candidate EV miRNAs, we discovered that miR‐95‐3p/miR‐26b‐5p had the highest AUC of 0.946 in the training cohort (Figure 4B). Below a cutoff of 0.06, the sensitivity to distinguish PDAC from CP patients was 86.7% and the specificity was 100.0% for the training cohort (Table 2). To validate the model performance, an independent cohort was tested. In the test cohort, the AUC for the miR‐95‐3p/miR‐26b‐5p was up to 0.875 (Additional file 1: Figure S4). Under the same cutoff of the quotient of the two miRNAs (miR‐95‐3p/miR‐26b‐5p), the sensitivity to distinguish PDAC from CP patients was 81.5% and the specificity was 93.3% (Figure 4C and Table 2). The prediction power in the test cohort was similar to that in the training cohort. In summary, the blood small EV miR‐95‐3p/miR‐26b‐5p showed an average sensitivity of 84.1% and an average specificity of 96.6% to identify PDAC from CP in the training cohort and the test cohort. Moreover, to validate the result of blood small EV miRNA miR‐95‐3p/miR‐26b‐5p experimentally, we used qRT‐PCR assay to confirm the effect of blood small EV miR‐95‐3p/miR‐26b‐5p on 24 PDAC and CP patients. The results of qRT‐PCR detection showed that the quotient of blood small EV miR‐95‐3p/miR‐26b‐5p could distinguish PDAC from CP patients (Additional file 1: Figure S5), which was similar to that results obtained using the HiSeq instrument and subsequent analysis (Figure 4C).

**TABLE 2:** Performance of the blood small EV miR‐95‐3p/miR‐26b‐5p in the training cohort and test cohort

	Predicted PDAC	Predicted CP	Sensitivity (%)	Specificity (%)
Training cohort				
PDAC (30)	26	4	86.7	100
CP (18)	0	18		
Test cohort				
PDAC (27)	22	5	81.5	93.3
CP (15)	1	14		

Abbreviations: CP, chronic pancreatitis; PDAC, pancreatic ductal adenocarcinoma.

After removing all of the metastatic PDAC specimens, the blood EV miRNAs miR‐95‐3p and miR‐26b‐5p found in this study were identified to be also capable of identifying nonmetastatic PDAC patients from CP patients in the training cohort and the test cohort (Additional file 1: Figure S6).

Since CA19‐9 has been reported as a blood‐based tumor marker for the management of pancreatic cancer, we calculated the AUC of CA19‐9 for distinguishing between PDAC and CP patients in this study. The AUC of CA19‐9 in the training cohort was 0.783 (Additional file 1: Figure S7), which was lower than the AUC of blood small EV miRNA miR‐95‐3p/miR‐26b‐5p (AUC = 0.948). The AUC of CA19‐9 in the test cohort was 0.796 (Additional file 1: Figure S7), which was also lower than the AUC of blood small EV miRNA miR‐95‐3p/miR‐26b‐5p (AUC = 0.875).

To improve the prediction power of the blood small EV miR‐95‐3p/miR‐26b‐5p, the prediction model was combined with the serum CA19‐9. The model is as follows:

$$f\;\left(x,y\right)=\left\{\begin{array}{c} \mathrm{Benign},\;x<0.06\;\mathrm{and}\;y<300\\ \mathrm{Tumor},\;\mathrm{else} \end{array}\right.$$

(x = miR‐95‐3p/miR‐26b‐5p; y = CA19‐9).

**FIGURE 5:**
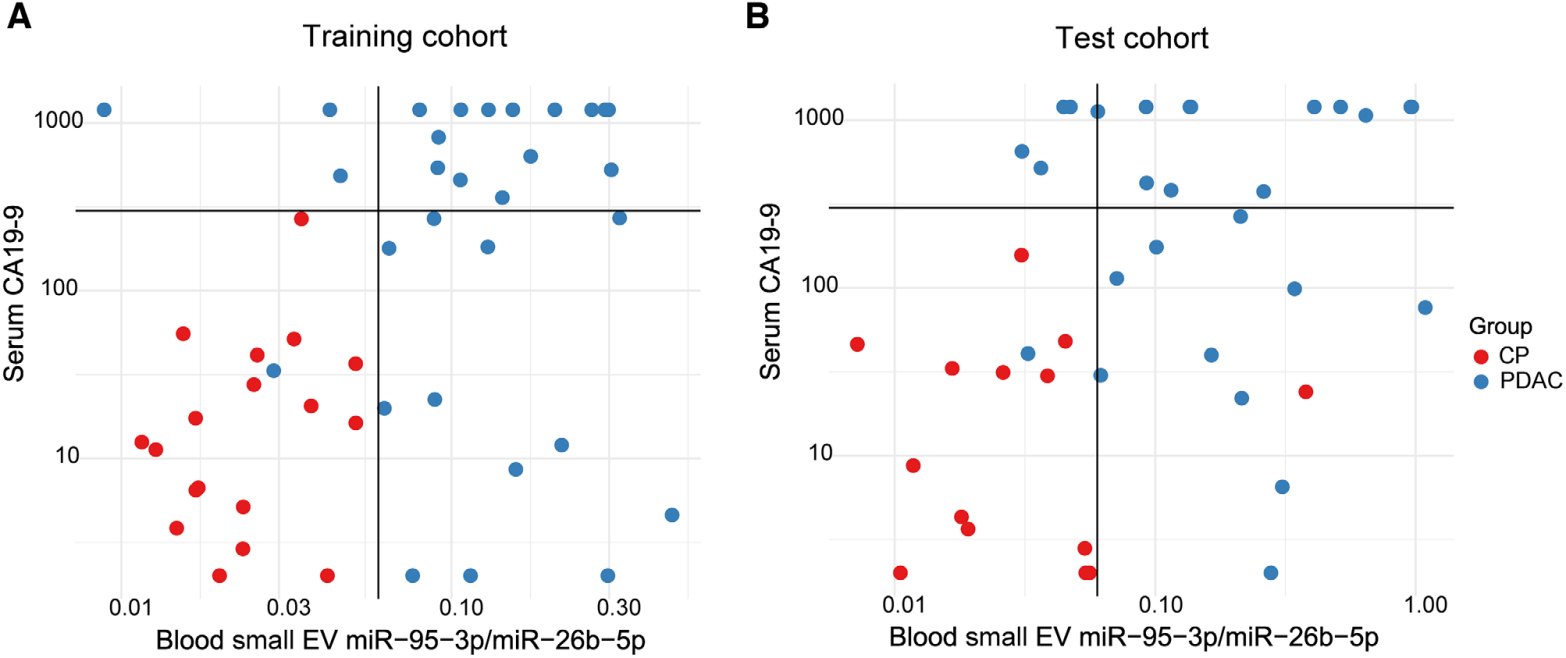
The blood small EV miR‐95‐3p/miR‐26b‐5p combined with the serum CA19‐9 to distinguish between PDAC and CP patients. (A) The distribution as scatter plots for the blood small EV miR‐95‐3p/miR‐26b‐5p and serum CA19‐9 in PDAC and CP from the training cohort. (B) The distribution as scatter plots for the blood small EV miR‐95‐3p/miR‐26b‐5p and serum CA19‐9 in PDAC and CP from the test cohort

In addition, the combination of the blood small EV miR‐95‐3p/miR‐26b‐5p and the serum CA‐19‐9 had superior performance compared with each model alone. When a specimen had either a serum level of CA19‐9 > 300 U/ml or a quotient of miR‐95‐3p/miR‐26b‐5p > 0.06 to be PDAC, the average sensitivity increased to 96.5% in both cohorts. In addition, the average specificity remained at 96.4% (Figure 5A, B and Table 3), which was a similar level when utilizing just the miR‐95‐3p/miR‐26b‐5p model alone.

**TABLE 3:** Performance of the blood small EV miR‐95‐3p/miR‐26b‐5p combined with serum levels of CA19‐9 in the training cohort and test cohort

	Predicted PDAC	Predicted CP	Sensitivity (%)	Specificity (%)
Training cohort				
PPDAC (30)	29	1	96.7	100
CP (18)	0	18		
Test cohort				
PPDAC (27)	26	1	96.3	92.8
CP (14)^a^	1	13		

^a^ Because 14 out of 15 patients had the expression data of both blood small EV miRNAs and serum CA19‐9 levels in the validation cohort, they were included.

Abbreviations: CP, chronic pancreatitis; PDAC, pancreatic ductal adenocarcinoma.

### 3.4 MiR‐95‐3pand miR‐26b‐5p were biologically associated with pancreatic cancer and pancreatitis processing

**FIGURE 6:**
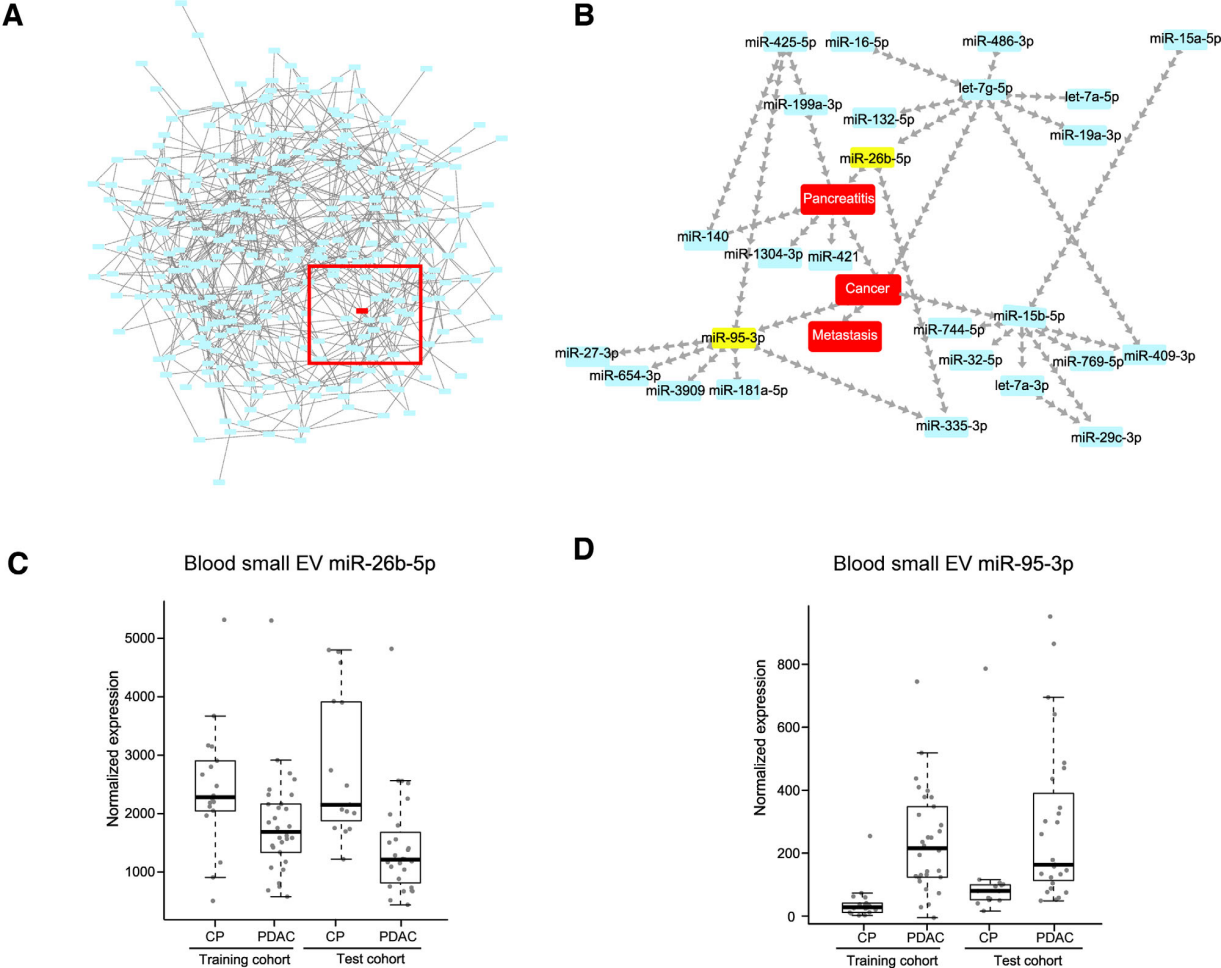
Biological correlations of the blood small EV miR‐95‐3p and miR‐26b‐5p with pancreatic cancer and pancreatitis. (A) The entire Bayesian network was constructed based on the blood small EV miRNA levels. (B) A subnetwork extracted from the entire Bayesian network, containing the “causes” and the consequences of the “cancer” node. (C) The distribution of levels showed as boxplots for the blood small EV miR‐26b‐5p in PDAC and CP from the training cohort and test cohort. (D) The distribution of levels shown as boxplots for the blood small EV miR‐95‐3p in PDAC and CP from the training cohort and test cohort

To further study the association of miR‐95‐3p and miR‐26b‐5p with pancreatic cancer and pancreatitis, a pathway enrichment analysis was performed on the experimental validated or predicted targets (Additional file 2: Table S6). However, their predicted or validated targets were enriched in lysine degradation, nucleotide excision repair, and other processes (Additional file 1: Figure S8), which did not clearly demonstrate their biological association with pancreatic cancer and pancreatitis. The Bayesian network is based on the probability of events and models, the causes and consequences of phenomena, and could potentially illustrate the connections between miRNA and cancer. In addition to discretized miRNA levels, three more factors were manually inserted to describe the specimen pathology, namely, “cancer” (for patients with PDAC), “pancreatitis” (for patients with pancreatitis), and “metastasis” (for patients with metastatic PDAC). The entire Bayesian network comprised 337 nodes and 713 edges (Figure 6A). From the whole network, a subnetwork, including the “cancer” node, and the causes and consequences of the “cancer” nodes were extracted (Figure 6B). Interestingly, we discovered that miR‐95‐3p was one of the “consequences” of cancer, and miR‐26b‐5p was one of the “causes” of pancreatitis (Figure 6B). Moreover, miR‐26b‐5p and miR‐95‐3p had the opposite trend of dysregulation in PDAC and CP patients (Figure 6C, D). The Bayesian network constructed using the blood small EV miRNA levels revealed the biological association of miR‐95‐3p and miR‐26b‐5p with pancreatic cancer and pancreatitis, further confirming the validity of the prediction model of the blood small EV miR‐95‐3p and miR‐26b‐5p to identify PDAC from CP patients.

### 3.5 Blood small EV miR‐335‐5p combined with miR‐340‐5p was identified as a potential metastasis prognostic biomarker

Among the 57 PDAC patients, there were 18 patients with nonmetastasis and 12 patients with metastasis in the training cohort, and 16 patients with nonmetastasis and 11 patients with metastasis in the test cohort (Table 1). A subgroup analysis on the potential metastatic biomarkers from blood small EV miRNAs was then conducted.

**FIGURE 7:**
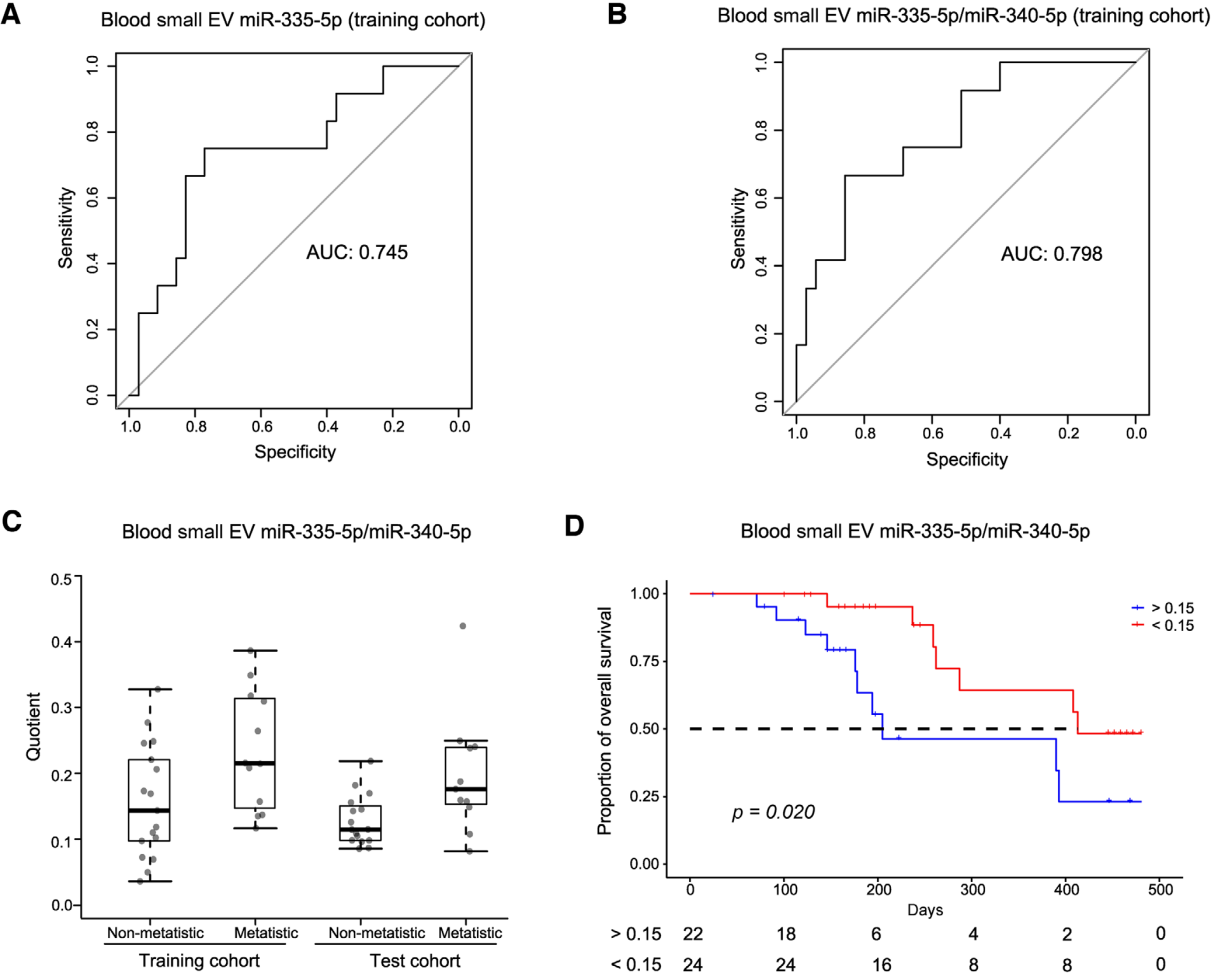
The blood small EV miR‐335‐5p/miR‐340‐5p was identified as a potential metastasis biomarker and able to reveal the prognosis. (A) The AUC of the ROC curves of the blood small EV miR‐335‐5p for distinguishing between metastasis patients and nonmetastasis patients in the training cohort is displayed. (B) The AUC of the ROC curve of the blood small EV miR‐335‐5p/miR‐340‐5p to differentiate between metastasis patients and nonmetastasis patients in the training cohort is displayed. (C) The distribution is shown as a boxplot for the quotients of the blood small EV miR‐335‐5p/miR‐340‐5p in patients from the training cohort and the test cohort with metastasis and nonmetastasis. (D) The Kaplan–Meier plot on the quotient of the blood small EV miR‐335‐5p/miR‐340‐5p is shown

Similar to the study of differential diagnosis biomarkers between PDAC and CP patients, the metastatic prediction of each miRNA was performed using the normalized expression with several filter conditions (miRNA expressed in all specimens; mean value of miRNA normalized expression ≥ 50; *p <* 0.05), and was also measured using AUC of the ROC curve. Additionally, due to the close association between the metastasis and the prognosis in pancreatic cancer,^34^ the selected candidate blood small EV miRNAs were demanded to be significantly correlated with survival. The results showed that among all of the candidate blood small EV miRNAs for differentiating between metastatic and nonmetastatic PDAC patients in the training cohort (Additional file 2: Table S7), miR‐335‐5p had the highest AUC of 0.745 (Figure 7A). Tumor tissue miRNA expressions of pancreatic cancer from TCGA revealed that miR‐335‐5p was significantly related to survival in pancreatic cancer (*p =* 0.033, Additional file 1: Figure S9). Moreover, we calculated the prediction AUC of miR‐335‐5p divided by all of the miRNAs in the training cohort (Additional file 2: Table S8). Among top five paired candidate miRNAs sorted by AUC, we found that only candidate partner miR‐340‐5p was significantly related to survival of pancreatic cancer in TCGA (*p =* 0.031, Additional file 1: Figure S10 and Additional file 2: Table S9). Therefore, we selected miR‐335‐5p/miR‐340‐5p with the AUC of 0.798 as the diagnostic biomarker for distinguishing between metastasis PDAC and nonmetastasis PDAC in the training cohort (Figure 7B). In the test cohort, the AUC of miR‐335‐5p/miR‐340‐5p for differential diagnosis between metastatic and nonmetastatic PDAC patients was up to 0.801 (Additional file 1: Figure S11). The quotient of miR‐335‐5p and miR‐340‐5p provided an accurate prediction and stable levels in the two cohorts (Figure 7C). In both cohorts, 46 patients had received surgery, and their OS after surgery could be evaluated (Additional file 2: Table S2). Patients with values above 0.15 of miR‐335‐5p/miR‐340‐5p had a worse OS (median OS, 205 days) than those with values below 0.15 (median OS, 413 days), with a log‐rank *p* value equal to 0.020 (Figure 7D). These preliminary results revealed that blood small EV miR‐335‐5p/miR‐340‐5p may be utilized as a potential metastasis prognostic PDAC biomarker.

## 4 DISCUSSION

This was the first report on the use of small RNA sequencing to identify the blood small EV‐derived miRNAs to distinguish PDAC from CP in clinical patients. The previous studies primarily focused on well‐studied miRNAs on microarrays and real‐time PCR platforms.^35^, ^36^, ^37^, ^38^ The first small RNA sequencing to detect exosomal miRNA reported by Ko et al. was from mice pancreatic cancer models.^39^ In this study, small RNA sequencing and clinical blood specimens from pathologically confirmed patients were used to identify blood small EV miR‐95‐3p/miR‐26b‐5p as a novel diagnostic biomarker to separate PDAC from CP patients.

In the present study, the sensitivity and specificity of blood small EV miR‐95‐3p/miR‐26b‐5p to differentiate PDAC from CP patients was as high as 86.7% and 100% for the training cohort, and 81.5% and 93.3% for the test cohort, respectively. This result demonstrated a better effect compared with other methods applied for differential diagnostics of pancreatic cancer, such as serum CA19‐9 (sensitivity = 79.0%, specificity = 82.0%),^8^ plasma miRNAs (sensitivity = 64.5–93.2%, specificity = 61.1–72.2%),^40^, ^41^ and ENS‐FNA (sensitivity = 53.0–73.9%, specificity = 73.7–100%).^5^ When blood small EV miR‐95‐3p/miR‐26b‐5p was combined with serum levels of CA19‐9, the average sensitivity was further enhanced to 96.5% in both cohorts, while the specificity was maintained at similar levels as the specificity of miR‐95‐3p/miR‐26b‐5p alone, which had better performance compared with previous studies.^4^, ^40^ The clinical effects of the blood small EV miR‐95‐3p/miR‐26b‐5p combined with the CA19‐9 serum levels to differentiate PDAC from CP patients still require a larger number of clinical specimens to validate these results.

Additionally, the biological relationship between miR‐95‐3p and miR‐26b‐5p and cancer was also investigated. Previous studies have reported that miR‐95‐3p can promote tumorigenesis of hepatocellular carcinoma and the development of prostatic cancer ^42^, ^43^ and the downregulation of miR‐95‐3p inhibits proliferation, in addition to promoting the apoptosis of glioma cells.^44^ In addition, miR‐26b‐5p has been reported to suppress the proliferation and promote apoptosis in multiple myeloma cells^45^ and regulate proliferation, angiogenesis, apoptosis, invasion, and metastasis in hepatocellular carcinoma.^46^, ^47^ Additionally, miR‐26b‐5p was found to be a tumor suppressor in papillary thyroid cancer^48^ and bladder cancer.^49^, ^50^ However, the functionality of miR‐95‐3p and miR‐26b‐5p in pancreatic cancer has not been reported. To further understand the biological association of miR‐95‐3p and miR‐26b‐5p with pancreatic cancer and pancreatitis, the Bayesian network was used to reveal the biological connections of blood small EV miRNAs with cancer and pancreatitis, which revealed that miR‐26b‐5p was the upstream miRNA of “pancreatitis” and miR‐95‐3p was the downstream miRNA of “cancer.” This biological association analysis further confirmed the combination of the blood small EV, miR‐95‐3p and miR‐26b‐5p, as a potential biomarker to distinguish PDAC from CP.

At present, metastatic pancreas cancers are mainly evaluated through medical imaging. However, these examinations have limited ability to detect small metastatic lesions.^51^ Some studies have reported that micrometastases in PDAC, which are difficult to detect using medical imaging or even surgery, can cause tumor recurrence within a few months after surgery.^52^, ^53^ Therefore, the diagnostic biomarkers to accurately distinguish metastatic from nonmetastatic PDAC are essential. A subgroup analysis identified for the first time that the blood small EV miR‐335‐5p/miR‐340‐5p could separate metastasis PDACs from nonmetastasis PDACs. Previous studies have reported that the downregulation of miR‐335‐5p can promote the migration and invasion of neuroblastoma.^54^ In addition, the upregulation of miR‐340‐5p can inhibit colorectal cancer cell proliferation, migration, and invasion.^55^ Moreover, tumor tissue miRNA expressions of pancreatic cancer from the TCGA revealed that both miR‐335‐5p and miR‐340‐5p were significantly related to survival of pancreatic cancer (Additional file 1: Figures S9 and S10). These functional studies and survival analyses supported the potential validity of the blood small EV miR‐335‐5p/miR‐340‐5p to distinguish metastasis from nonmetastasis PDAC.

In this study, a blood small EV miRNA model was established and validated for assisting physicians to differentiate PDAC from CP. Participants who could not be diagnosed and were identified as PDAC or CP detected using imaging detection were further stratified according to the results of the blood small EV miRNA model in the clinic. CP patients predicted using this model to have low risk may be able to just undergo the typical follow‐up. However, patients predicted by this model to be high risk for PDAC should be further evaluated by a physician to make further decisions.

This study had several limitations. First, biological functions and downstream genes of the blood small EV miRNA biomarkers were unclear in pancreatic cancer and need to be explored in the next research step. Second, although the blood small EV miRNAs show promise as a liquid biopsy that is minimally invasive, convenient, and capable of repeatable sampling, the blood small EV miRNAs were detected using small RNA sequencing in this study, which takes about 6 days and costs nearly 350 USD for a single sample and is not readily applicable in clinical *in vitro* diagnostic detections. Thus, other applicable methods for the detection of blood small EV miRNAs, such as Q‐PCR and chips, need to be tested. Third, although it was discovered and preliminarily validated that the blood small EV miRNAs could be used as differential diagnosis and metastasis biomarkers for PDAC, a prospective study with a large number of clinical samples, including an external cohort, is still required to confirm the clinical effects of the blood small EV miRNAs.

## 5 CONCLUSION

In summary, this study reported the profiles of blood small EV miRNAs in the patients with PDAC or CP. Moreover, it was found that the blood small EV miR‐95‐3p/miR‐26b‐5p and its combination with serum CA19‐9 levels could distinguish between PDAC and CP. Further biological associations of miR‐95‐3p and miR‐26b‐5p with pancreatic cancer and pancreatitis confirmed the validity of the prediction model. In addition, subgroup analysis revealed that the blood small EV miR‐335‐5p/miR‐340‐5p was identified to associate with PDAC metastasis and poor prognosis. The results demonstrated that blood small EV miRNAs can be utilized as a new promising biomarker to assist with the differential diagnosis and metastasis prognosis of PDAC.

## CONFLICT OF INTEREST

All authors affiliated with 3D Medicines Inc. are current or former employees. No potential conflict of interest was disclosed by the other authors.

## ETHICS APPROVAL AND CONSENT TO PARTICIPATE

All patients enrolled signed informed consent. This study was approved by institutional review board (CHEC2018‐039).

## AUTHORCONTRIBUTIONS

GJ, XJ, and DZ presented the concept. GJ, XJ, DZ, SG, HQ, and SB conceived and designed the experiments. SG, HQ, KL, HW, SL, SB, ZS, BS, XX, JS, PZ, XS, SG, JX, and YP performed the experiments. SG, HQ, KL, HW, SL, DZ, XX, and FL analyzed the data. SG, KL, HW, SL, SB, YZ, HC, LX, DZ, XJ, and GJ contributed to reagents/material/data interpretation. DZ, HQ, SL, and SG helped with manuscript writing. DZ, KL, and XJ helped with manuscript revision. All authors read and approved the final manuscript.

## Supporting information

SUPPORTING MATERIAL

SUPPORTING MATERIAL

SUPPORTING MATERIAL

SUPPORTING MATERIAL

SUPPORTING MATERIAL

SUPPORTING MATERIAL

SUPPORTING MATERIAL

SUPPORTING MATERIAL

SUPPORTING MATERIAL

SUPPORTING MATERIAL

SUPPORTING MATERIAL

## Data Availability

The datasets used during the current study are available from the corresponding author on reasonable request.

## References

[1] Siegel RL, Miller KD, Jemal A. Cancer statistics. CA Cancer J Clin. 2018;68:7‐30. 29313949 10.3322/caac.21442

[2] Peery AF, Dellon ES, Lund J, et al. Burden of gastrointestinal disease in the United States: 2012 update. Gastroenterology. 2012;143:e3. 10.1053/j.gastro.2012.08.002 PMC3480553 22885331

[3] Kirkegard J, Mortensen FV, Cronin‐Fenton D. Chronic pancreatitis and pancreatic cancer risk: a systematic review and meta‐analysis. Am J Gastroenterol. 2017;112:1366‐1372. 28762376 10.1038/ajg.2017.218

[4] Mayerle J, Kalthoff H, Reszka R, et al. Metabolic biomarker signature to differentiate pancreatic ductal adenocarcinoma from chronic pancreatitis. Gut. 2018;67:128‐137. 28108468 10.1136/gutjnl-2016-312432 PMC5754849

[5] Dutta AK, Chacko A. Head mass in chronic pancreatitis: inflammatory or malignant. World J Gastrointest Endosc. 2015;7:258‐264. 25789097 10.4253/wjge.v7.i3.258 PMC4360445

[6] Kato K, Nihashi T, Ikeda M, et al. Limited efficacy of (18)F‐FDG PET/CT for differentiation between metastasis‐free pancreatic cancer and mass‐forming pancreatitis. Clin Nucl Med. 2013;38:417‐421. 23486318 10.1097/RLU.0b013e3182817d9d

[7] Minaga K, Takenaka M, Katanuma A, et al. Needle tract seeding: an overlooked rare complication of endoscopic ultrasound‐guided fine‐needle aspiration. Oncology. 2017; 93(Suppl):107‐112. 29258068 10.1159/000481235

[8] Goonetilleke KS, Siriwardena AK. Systematic review of carbohydrate antigen (CA 19‐9) as a biochemical marker in the diagnosis of pancreatic cancer. Eur J Surg Oncol. 2007;33:266‐270. 17097848 10.1016/j.ejso.2006.10.004

[9] Poruk KE, Gay DZ, Brown K, et al. The clinical utility of CA 19‐9 in pancreatic adenocarcinoma: diagnostic and prognostic updates. Curr Mol Med. 2013;13:340‐351. 23331006 10.2174/1566524011313030003 PMC4419808

[10] He C, Zheng S, Luo Y, et al. Exosome theranostics: biology and translational medicine. Theranostics. 2018;8:237‐255. 29290805 10.7150/thno.21945 PMC5743472

[11] ELA S, Mager I, Breakefield XO, et al. Extracellular vesicles: biology and emerging therapeutic opportunities. Nat Rev Drug Discov. 2013;12:347‐357. 23584393 10.1038/nrd3978

[12] Nakamura S, Sadakari Y, Ohtsuka T, et al. Pancreatic juice exosomal microRNAs as biomarkers for detection of pancreatic ductal adenocarcinoma. Ann Surg Oncol. 2019;26:2104‐2111. 30820789 10.1245/s10434-019-07269-z

[13] Ciardiello C, Cavallini L, Spinelli C, et al. Focus on extracellular vesicles: new frontiers of cell‐to‐cell communication in cancer. Int J Mol Sci. 2016;17:175. 26861306 10.3390/ijms17020175 PMC4783909

[14] Hannafon BN, Ding WQ. Intercellular communication by exosome‐derived microRNAs in cancer. Int J Mol Sci. 2013;14:14240‐14269. 23839094 10.3390/ijms140714240 PMC3742242

[15] Jin X, Chen Y, Chen H, et al. Evaluation of tumor‐derived exosomal miRNA as potential diagnostic biomarkers for early‐stage non‐small cell lung cancer using next‐generation sequencing. Clin Cancer Res. 2017;23:5311‐5319. 28606918 10.1158/1078-0432.CCR-17-0577

[16] Wang M, Ji S, Shao G, et al. Effect of exosome biomarkers for diagnosis and prognosis of breast cancer patients. Clin Transl Oncol. 2018;20:906‐911. 29143228 10.1007/s12094-017-1805-0

[17] Zhao L, Yu J, Wang J, et al. Isolation and identification of miRNAs in exosomes derived from serum of colon cancer patients. J Cancer. 2017;8:1145‐1152. 28607588 10.7150/jca.18026 PMC5463428

[18] Kawamura S, Iinuma H, Wada K, et al. Exosome‐encapsulated microRNA‐4525, microRNA‐451a and microRNA‐21 in portal vein blood is a high‐sensitive liquid biomarker for the selection of high‐risk pancreatic ductal adenocarcinoma patients. J Hepatobiliary Pancreat Sci. 2019;26:63‐72. 30561106 10.1002/jhbp.601

[19] Pang Y, Wang C, Lu L, et al. Dual‐SERS biosensor for one‐step detection of microRNAs in exosome and residual plasma of blood samples for diagnosing pancreatic cancer. Biosens Bioelectron. 2019;130:204‐213. 30745282 10.1016/j.bios.2019.01.039

[20] Goto T, Fujiya M, Konishi H, et al. An elevated expression of serum exosomal microRNA‐191, ‐ 21, ‐451a of pancreatic neoplasm is considered to be efficient diagnostic marker. BMC Cancer. 2018;18:116. 29385987 10.1186/s12885-018-4006-5 PMC5793347

[21] Que R, Ding G, Chen J, et al. Analysis of serum exosomal microRNAs and clinicopathologic features of patients with pancreatic adenocarcinoma. World J Surg Oncol. 2013;11:219. 24007214 10.1186/1477-7819-11-219 PMC3766671

[22] Xu YF, Hannafon BN, Zhao YD, et al. Plasma exosome miR‐196a and miR‐1246 are potential indicators of localized pancreatic cancer. Oncotarget. 2017;8:77028‐77040. 29100367 10.18632/oncotarget.20332 PMC5652761

[23] Lai X, Wang M, McElyea SD, et al. A microRNA signature in circulating exosomes is superior to exosomal glypican‐1 levels for diagnosing pancreatic cancer. Cancer Lett. 2017;393:86‐93. 28232049 10.1016/j.canlet.2017.02.019 PMC5386003

[24] Linda DM, Andreas PN, Julia SJ, et al. Serum biomarker signature‐based liquid biopsy for diagnosis of early‐stage pancreatic cancer. J Clin Oncol. 2018;36:2887‐2894. 30106639 10.1200/JCO.2017.77.6658 PMC6161836

[25] Darwin LC, Linda SL, Dhiraj Y, et al. American Pancreatic Association Practice Guidelines in Chronic Pancreatitis: evidence‐based report on diagnostic guidelines. Pancreas. 2014;43:1143‐1162. 25333398 10.1097/MPA.0000000000000237 PMC5434978

[26] Zhang JT, Qin H, Cheung FKM, et al. Plasma extracellular vesicle microRNAs for pulmonary ground‐glass nodules. J Extracell Vesicles. 2019;8:1663666. 31579436 10.1080/20013078.2019.1663666 PMC6758624

[27] Li H, Durbin R. Fast and accurate short read alignment with Burrows–Wheeler transform. Bioinformatics. 2009;25:1754‐1760. 19451168 10.1093/bioinformatics/btp324 PMC2705234

[28] Griffiths‐Jones S, Saini HK, van Dongen S, et al. miRBase: tools for microRNA genomics. Nucleic Acids Res. 2008;36:D154‐D158. 17991681 10.1093/nar/gkm952 PMC2238936

[29] Love MI, Huber W, Anders S. Moderated estimation of fold change and dispersion for RNA‐seq data with DESeq2. Genome Biol. 2014;15:550. 25516281 10.1186/s13059-014-0550-8 PMC4302049

[30] Vlachos IS, Zagganas K, Paraskevopoulou MD, et al. DIANA‐miRPath v3.0: deciphering microRNA function with experimental support. Nucleic Acids Res. 2015;43:W460‐W466. 25977294 10.1093/nar/gkv403 PMC4489228

[31] Anaya J. OncoLnc: linking TCGA survival data to mRNAs, miRNAs, and lncRNAs. PeerJ Comp Sci. 2016;2:e67.

[32] Thery C, Witwer KW, Aikawa E, et al. Minimal information for studies of extracellular vesicles 2018 (MISEV2018): a position statement of the International Society for Extracellular Vesicles and update of the MISEV2014 guidelines. J Extracell Vesicles. 2018;7:1535750. 30637094 10.1080/20013078.2018.1535750 PMC6322352

[33] Fotuhi SN, Khalaj‐Kondori M, Feizi MAH, et al. Long non‐coding RNA BACE1‐AS may serve as an Alzheimer's disease blood‐based biomarker. J Mol Neurosci. 2019;69:351‐359. 31264051 10.1007/s12031-019-01364-2

[34] Peixoto RD, Speers C, McGahan CE, et al. Prognostic factors and sites of metastasis in unresectable locally advanced pancreatic cancer. Cancer Med. 2015;4:1171‐1177. 25891650 10.1002/cam4.459 PMC4559028

[35] Muller S, Raulefs S, Bruns P, et al. Next‐generation sequencing reveals novel differentially regulated mRNAs, lncRNAs, miRNAs, sdRNAs and a piRNA in pancreatic cancer. Mol Cancer. 2015;14:94. 25910082 10.1186/s12943-015-0358-5 PMC4417536

[36] Johansen JS, Calatayud D, Albieri V, et al. The potential diagnostic value of serum microRNA signature in patients with pancreatic cancer. Int J Cancer. 2016;139:2312‐2324. 27464352 10.1002/ijc.30291

[37] Ali S, Dubaybo H, Brand RE, et al. Differential expression of microRNAs in tissues and plasma co‐exists as a biomarker for pancreatic cancer. J Cancer Sci Ther. 2015;7:336‐346. 26819679 10.4172/1948-5956.1000372 PMC4725594

[38] Nakamura S, Sadakari Y, Ohtsuka T, et al. Pancreatic juice exosomal microRNAs as biomarkers for detection of pancreatic ductal adenocarcinoma. Ann Surg Oncol. 2019;26:2104‐2111. 30820789 10.1245/s10434-019-07269-z

[39] Ko J, Bhagwat N, Black T, et al. miRNA profiling of magnetic nanopore‐isolated extracellular vesicles for the diagnosis of pancreatic cancer. Cancer Res. 2018;78:3688‐3697. 29735554 10.1158/0008-5472.CAN-17-3703

[40] Liu J, Gao J, Du Y, et al. Combination of plasma microRNAs with serum CA19‐9 for early detection of pancreatic cancer. Int J Cancer. 2012;131:683‐691. 21913185 10.1002/ijc.26422

[41] Vychytilova‐Faltejskova P, Kiss I, Klusova S, et al. MiR‐21, miR‐34a, miR‐198 and miR‐217 as diagnostic and prognostic biomarkers for chronic pancreatitis and pancreatic ductal adenocarcinoma. Diagn Pathol. 2015;10:38. 25908274 10.1186/s13000-015-0272-6 PMC4407796

[42] Ye J, Yao Y, Song Q, et al. Up‐regulation of miR‐95‐3p in hepatocellular carcinoma promotes tumorigenesis by targeting p21 expression. Sci Rep. 2016;6:34034. 27698442 10.1038/srep34034 PMC5048429

[43] Xi M, Cheng L, Hua W, et al. MicroRNA‐95‐3p promoted the development of prostatic cancer via regulating DKK3 and activating Wnt/beta‐catenin pathway. Eur Rev Med Pharmacol Sci. 2019;23:1002‐1011. 30779066 10.26355/eurrev_201902_16987

[44] Fan B, Jiao BH, Fan FS, et al. Downregulation of miR‐95‐3p inhibits proliferation, and invasion promoting apoptosis of glioma cells by targeting CELF2. Int J Oncol. 2015;47:1025‐1033. 26165303 10.3892/ijo.2015.3080

[45] Jia CM, Tian YY, Quan LN, et al. miR‐26b‐5p suppresses proliferation and promotes apoptosis in multiple myeloma cells by targeting JAG1. Pathol Res Pract. 2018;214:1388‐1394. 30098829 10.1016/j.prp.2018.07.025

[46] Wang Y, Sun BC, Sun HZ, et al. Regulation of proliferation, angiogenesis and apoptosis in hepatocellular carcinoma by miR‐26b‐5p. Tumour Biol. 2016;37:10965‐10979. 26891666 10.1007/s13277-016-4964-7

[47] Wang Y, Sun BC, Zhao XL, et al. Twist1‐related miR‐26b‐5p suppresses epithelial‐mesenchymal transition, migration and invasion by targeting SMAD1 in hepatocellular carcinoma. Oncotarget. 2016;26:24383‐24401. 10.18632/oncotarget.8328 PMC5029709 27027434

[48] Zhou AY, Pan HY, Sun DJ, et al. miR‐26b‐5p inhibits the proliferation, migration and invasion of human papillary thyroid cancer in a β‐catenin‐dependent manner. Onco Targets Ther. 2020;13:1593‐1603. 32110056 10.2147/OTT.S236319 PMC7041607

[49] Miyamoto K, Seki N, Matsushita R, et al. Tumour‐suppressive miRNA‐26a‐5p and miR‐26b‐5p inhibit cell aggressiveness by regulating PLOD2 in bladder cancer. Br J Cancer. 2016;115:354‐363. 27310702 10.1038/bjc.2016.179 PMC4973152

[50] Wu K, Mu XY, Jiang JT, et al. miRNA‑26a‑5p and miR‑26b‑5p inhibit the proliferation of bladder cancer cells by regulating PDCD10. Oncol Rep. 2018;40:3523‐3532. 30272373 10.3892/or.2018.6734

[51] Iafrate F, Ciolina M, Sammartino P, et al. Peritoneal carcinomatosis: imaging with 64‐MDCT and 3T MRI with diffusion‐weighted imaging. Abdom Imaging. 2012;37:616‐627. 21972153 10.1007/s00261-011-9804-z

[52] Choi SB, Han HJ, Park P, et al. Systematic review of the clinical significance of lymph node micrometastases of pancreatic adenocarcinoma following surgical resection. Pancreatology. 2017;17:342‐349. 28336226 10.1016/j.pan.2017.03.008

[53] Katada T, Hashidate H, Yokoyama N, et al. Initial features of hepatic metastases from pancreatic cancer: histological and radiolographical appraisal of hepatic micrometastases detected by real‐time fluorescent imaging. Pancreas. 2017;46:1196‐1201. 28902791 10.1097/MPA.0000000000000915

[54] Lynch J, Fay J, Meehan M, et al. MiRNA‐335 suppresses neuroblastoma cell invasiveness by direct targeting of multiple genes from the non‐canonical TGF‐beta signalling pathway. Carcinogenesis. 2012;33:976‐985. 22382496 10.1093/carcin/bgs114 PMC3334516

[55] Yang L, Men WL, Yan KM, et al. MiR‐340‐5p is a potential prognostic indicator of colorectal cancer and modulates ANXA3. Eur Rev Med Pharmacol Sci. 2018;22:4837‐4845. 30070320 10.26355/eurrev_201808_15619

