# Supplementary material for: Blood small extracellular vesicles derived miRNAs to differentiate pancreatic ductal adenocarcinoma from chronic pancreatitis
Source: Clin Transl Med. 2021 Sep 10;11(9):e520. doi: 10.1002/ctm2.520 (PMC8431442; doi:10.1002/ctm2.520)
Supplement: Supplementary file 1 — SUPPORTING MATERIAL [file CTM2-11-e520-s005.pdf]

Additional file 1

Blood small extracellular vesicles derived miRNAs to differentiate pancreatic ductal adenocarcinoma from chronic pancreatitis

Shiwei Guo[1†], Hao Qin[2†], Ke Liu[3†], Huan Wang[1†], Shiyi Liu[2†], Sijia Bai[1†], Zhuo Shao[1], Yanan Zhang[2], Bin Song[1], Xiaoya Xu[2], Jing Shen[1], Peng Zeng[2], Xiaohan Shi[1], Hao Chen[2], Suizhi Gao[1], Jiajia Xu[2], Yaqi Pan[1], Lei Xiong[2], Fugen Li[2], Dadong Zhang[2*], Xiaodong Jiao[3*], Gang Jin[1*]

[1]Department of Hepatobiliary Pancreatic Surgery, Changhai Hospital, Navy Military Medical University, Shanghai 200433, China

[2]3D Medicines Inc., Shanghai 201114, China

[3]Department of Medical Oncology, Changzheng Hospital, Shanghai 200070, China

[†]Shiwei Guo, Hao Qin, Ke Liu, Huan Wang, Shiyi Liu and Sijia Bai contributed equally to this work.

[*]Co-corresponding authors.

Additional file 1：FigureS1. The flowchart of this study.
Additional file 1：FigureS2. Blood small EV profiles on the PC1 and PC2 in the healthy population as well as PC and PDAC patients from the test cohort.
Additional file 1：FigureS3. The profiles of blood small EV miRNAs between CP patients and PDAC patients in the training cohort and the test cohort.
Additional file 1：Figure S4. Blood small EV miR-95-3p/miR-26b-5p to distinguish between PDAC and CP patients in the test cohort.
Additional file 1：Figure S5. Validation results of blood small EV miR-95-3p/miR-26b-5p to distinguish between PDAC and CP using qRT-PCR.
Additional file 1：Figure S6. The quotient of blood small EV miR-95-3p over miR-26b-5p for distinguishing between non-metastatic PDAC and CP patients.
Additional file 1：Figure S7. The AUC of ROC curves of serum CA19-9 for distinguishing between PDAC and CP patients in the training cohort and the test cohort.
Additional file 1：Figure S8. Pathway enrichment analysis of the mRNA targets of candidate blood small EV miRNAs.
Additional file 1：Figure S9. Survival curves of samples with different expression levels of miR-335-5p usingTCGA data.
Additional file 1：Figure S10. Survival curves of samples with different expression levels of miR-340-5p usingTCGA data.
Additional file 1：Figure S11. Blood small EV miR-335-5p/miR-340-5p to distinguish between metastasis patients and non-metastasis in the test cohort.

Additional file 1：Figure S1

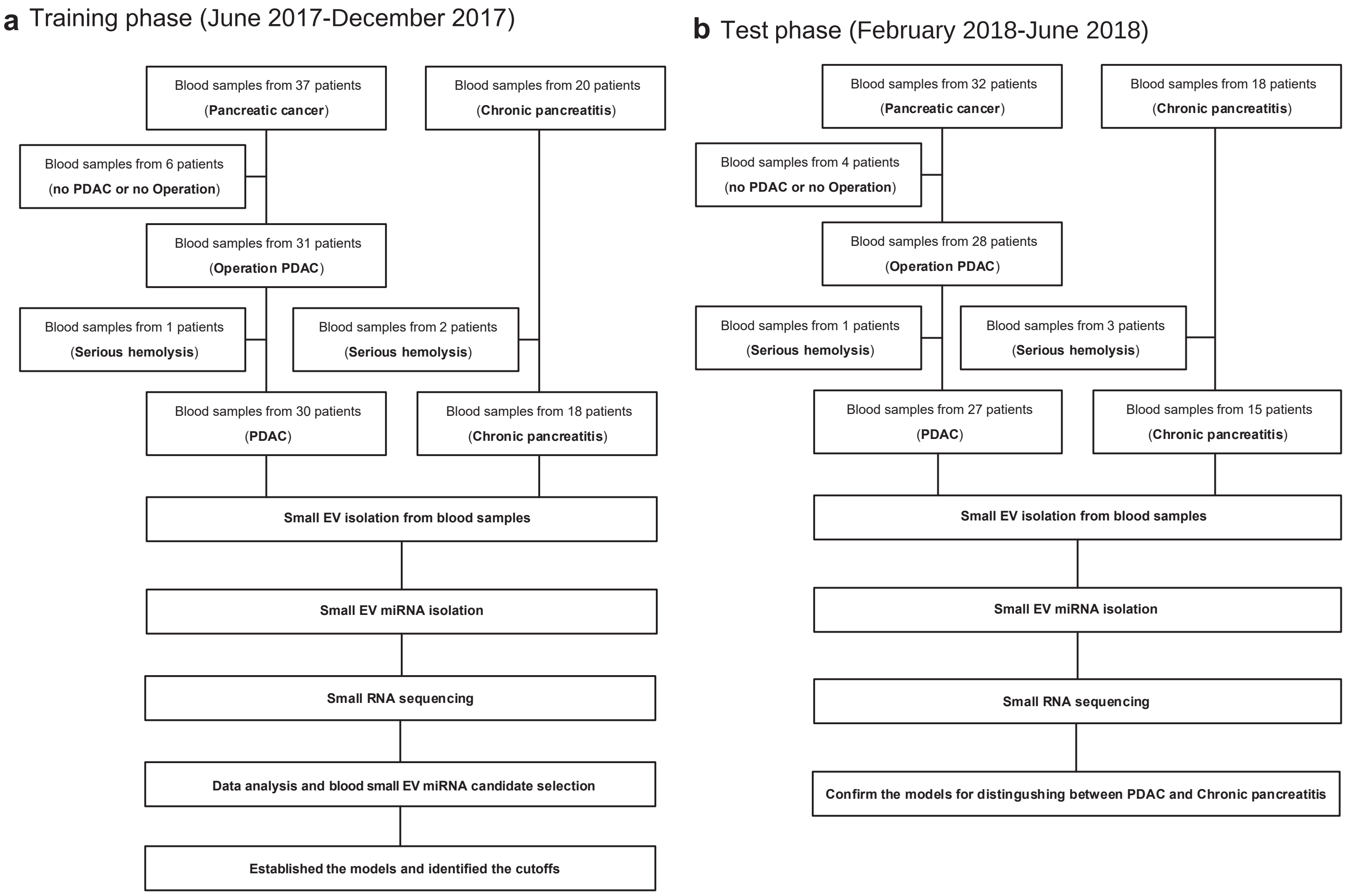

Additional file 1：Figure S1. The flowchart of this study.

a Training phase began with selecting PDAC patients (n = 37) and CP patients (n = 20), continuing with elimination of pathology samples from patients with non-PDAC, no operation and serious hemolysis. EVs were isolated from blood samples of 30 PDAC patients and 18 CP patients. Small RNA sequencing were used to detect the expression levels of blood EV miRNAs and select the miRNA candidates in the training cohort (n = 48), in order to establish the models and identify the cutoffs. **b** For the test phase, PDAC patients (n = 32) and CP patients (n = 18) was selected and small RNA sequencing was carried out in the test cohort (n = 42) in order to confirm model for distinguishing between PDAC and CP.

Additional file 1：Figure S2

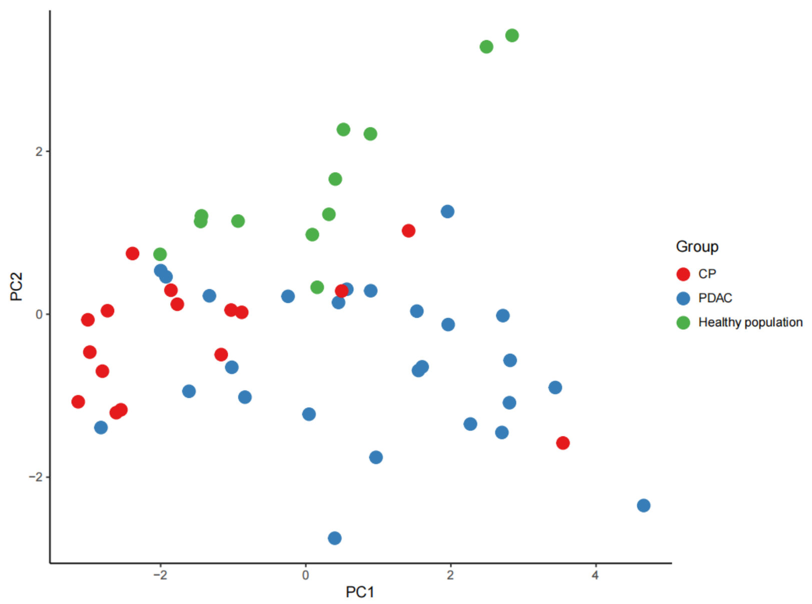

Additional file 1：Figure S2. Blood small EV profiles on the PC1 and PC2 in the healthy pop ulation as well as PC and PDAC patients from the test cohort.

Population groups (Health participants, PDAC patients and CP patients) are denoted by color.

Additional file 1：Figure S3

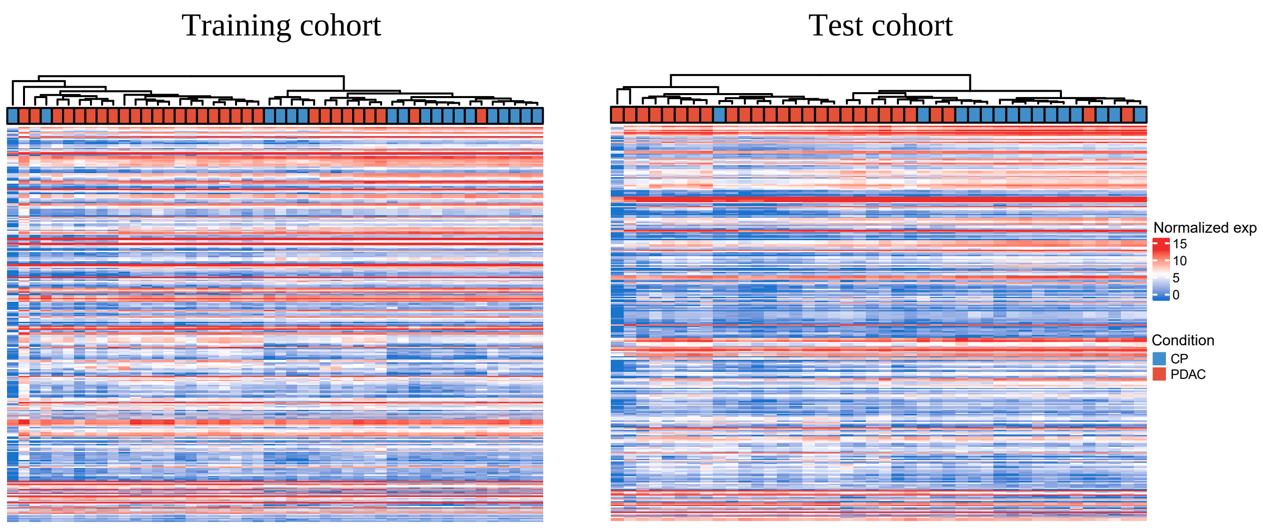

Additional file 1：Figure S3. The profiles of blood small EV miRNAs between CP patients and PDAC patients in the training cohort and the test cohort.

The heatmaps are shown for the profiles of the blood small EV miRNAs in the training cohort and the test cohort.

Additional file 1：Figure S4

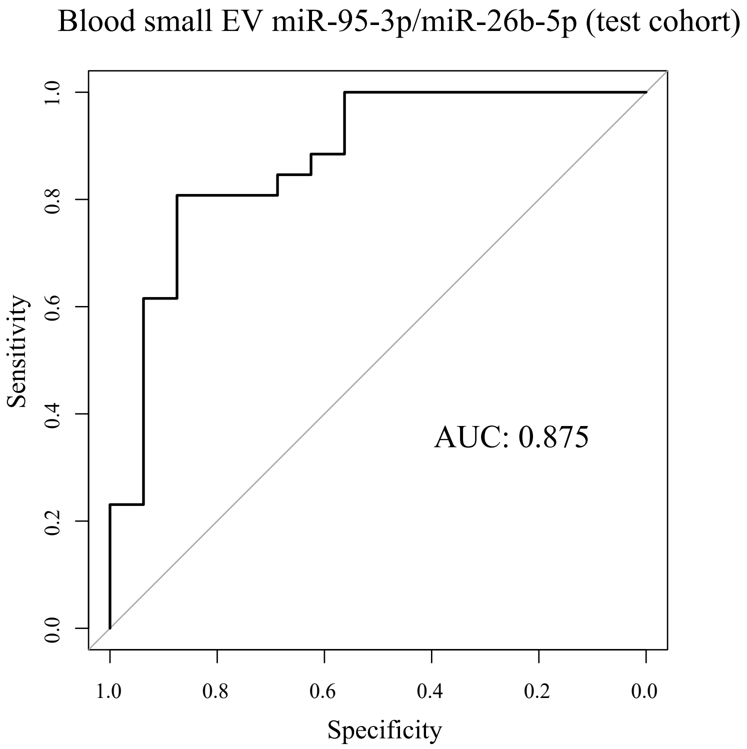

Additional file 1：Figure S4. Blood small EV miR-95-3p/miR-26b-5p to distinguish between PDAC and CP patients in the test cohort.
The AUC of the ROC curve of blood small EV miR-95-3p/miR-26b-5p for distinguishing between PDAC and CP patients in the test cohort was displayed.

Additional file 1：Figure S5

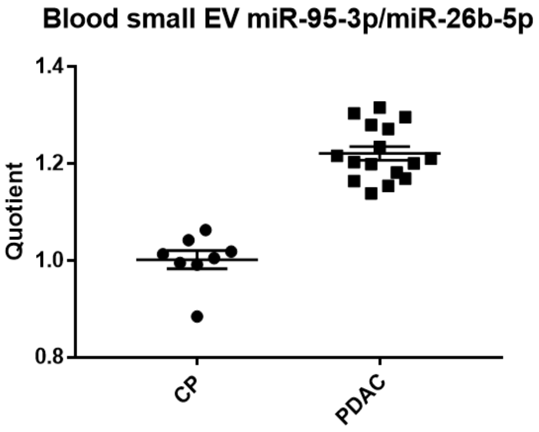

Additional file 1：Figure S5. Validation results of blood small EV miR-95-3p/miR-26b-5p to distinguish between PDAC and CP using qRT-PCR.
The distribution of levels were shown for the quotient of blood small EV miR-95-3p over miR-26b-5p detected using qRT-PCR for distinguishing between PDAC and CP in 24 clinical specimens.

Additional file 1：Figure S6

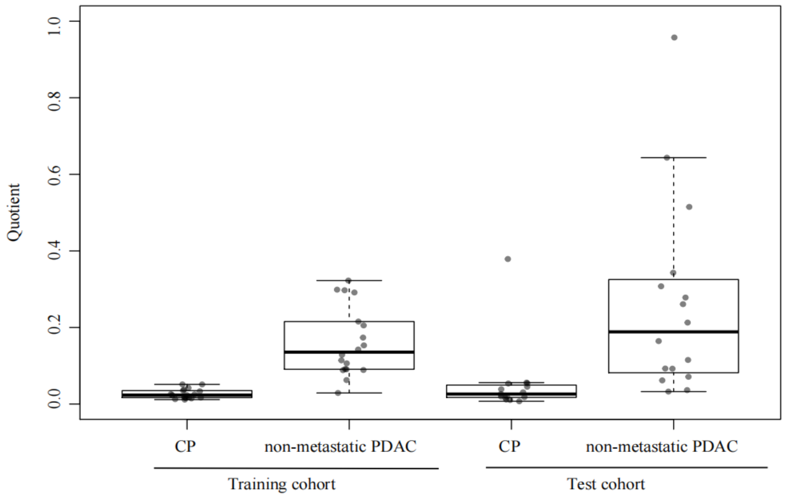

Additional file 1：Figure S6. The quotient of blood small EV miR-95-3p over miR-26b-5p for distinguishing between non-metastatic PDAC and CP patients.

The distribution of levels were shown as boxplots for the quotient of blood small EV miR-95-3p over miR-26b-5p for distinguishing between non-metastatic PDAC and CP patients in the training cohort and test cohort

Additional file 1：Figure S7

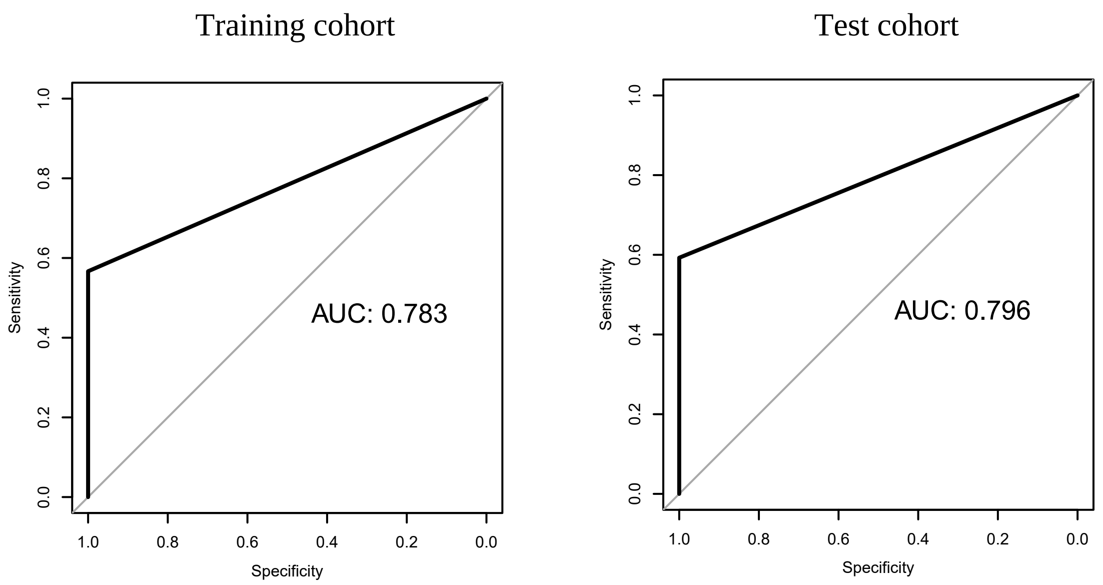

Additional file 1：Figure S7. The AUC of ROC curves of serum CA19-9 for distinguishing between PDAC and CP patients in the training cohort and the test cohort.

The AUC of the ROC curves of serum CA19-9 for distinguishing between PDAC and CP patients in the training cohort and the test cohort were displayed.

Additional file 1：Figure S8

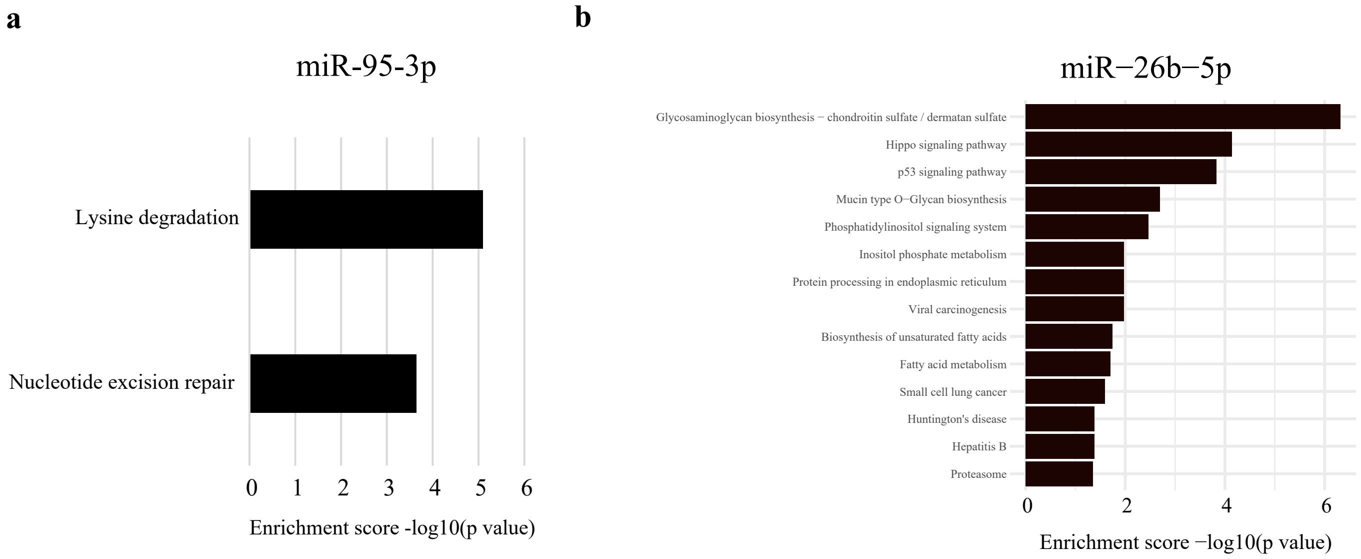

Additional file 1：Figure S8. Pathway enrichment analysis of the mRNA targets of candidate blood small EV miRNAs.

(a) The pathway enrichment of the predicted mRNA targets of miR-95-3p was displayed. (b) The pathway enrichment of experimental validated mRNA targets of miR-26b-5p was shown.

Additional file 1：Figure S9

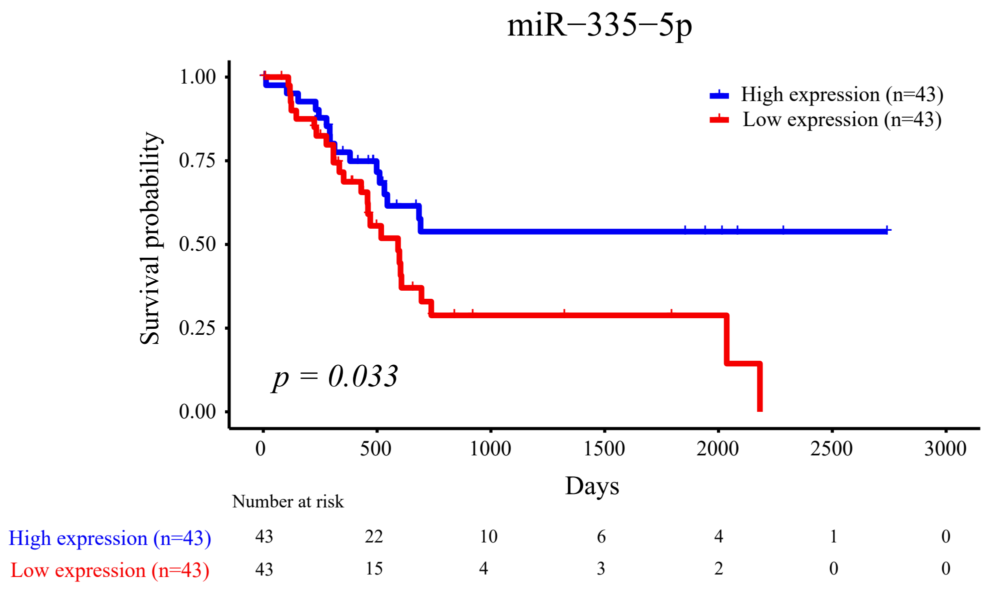

Additional file 1：Figure S9. Survival curves of samples with different expression levels of miR-335-5p using TCGA data.
Kaplan–Meier plot on the upper quarter and the lowest quarter levels of miR-335-5p in tumor tissues from TCGA.

Additional file 1：Figure S10

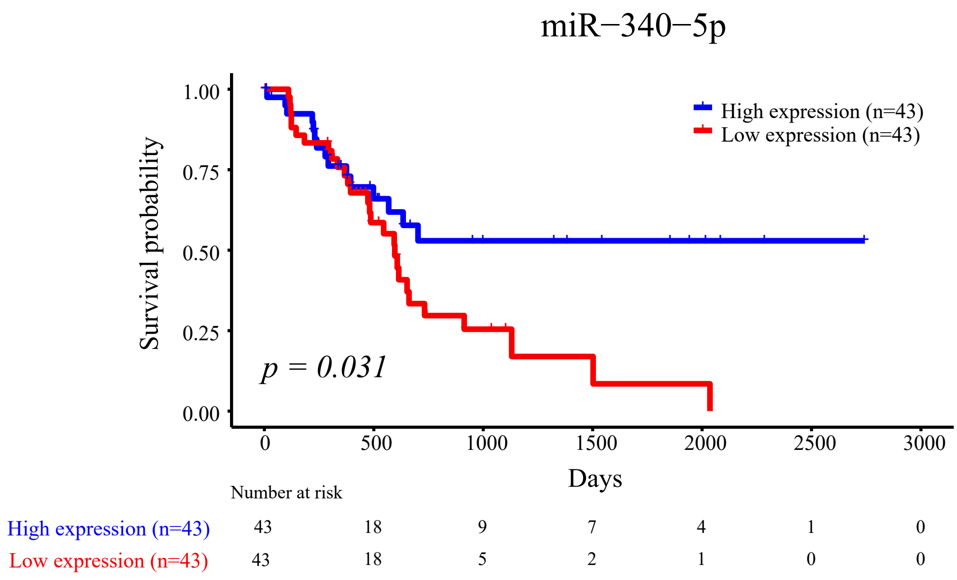

Additional file 1：Figure S10. Survival curves of samples with different expression levels of miR-340-5p using TCGA data.
Kaplan–Meier plot on the upper quarter and the lowest quarter levels of miR-340-5p in tumor tissues from TCGA.

Additional file 1：Figure S11

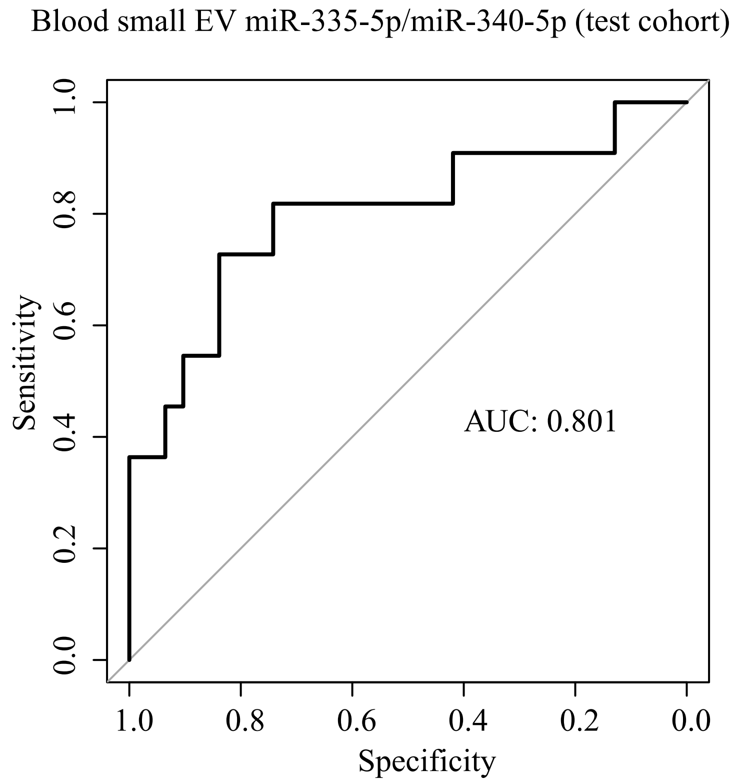

Additional file 1：Figure S11. Blood small EV miR-335-5p/miR-340-5p to distinguish between metastasis patients and non-metastasis in the test cohort.
The AUC of the ROC curve of blood small EV miR-335-5p/miR-340-5p for distinguishing between metastasis patients and non-metastasis patients in the test cohort was displayed.
