# Supplementary material for: Blood small extracellular vesicles derived miRNAs to differentiate pancreatic ductal adenocarcinoma from chronic pancreatitis
Source: Clin Transl Med. 2021 Sep 10;11(9):e520. doi: 10.1002/ctm2.520 (PMC8431442; doi:10.1002/ctm2.520)
Supplement: Supplementary file 2 — SUPPORTING MATERIAL [file CTM2-11-e520-s004.docx]

**Description of Additional file 1 and 2**

**Additional file 1：Figure S1**. The flowchart of this study. **Figure S2**. Blood small EV miR-95-3p/miR-26b-5p to distinguish between PDAC and CP patients in the test cohort. **Figure S3**. The profiles of blood small EV miRNAs between CP patients and PDAC patients in the training cohort and the test cohort. **Figure S4**. Blood small EV miR-95-3p/miR-26b-5p to distinguish between PDAC and CP patients in the test cohort. **Figure S5**. Validation results of blood small EV miR-95-3p/miR-26b-5p to distinguish between PDAC and CP using qRT-PCR. **Figure S6**. The quotient of blood small EV miR-95-3p over miR-26b-5p for distinguishing between non-metastatic PDAC and CP patients. **Figure S7**. The AUC of ROC curves of serum CA19-9 for distinguishing between PDAC and CP patients in the training cohort and the test cohort. **Figure S8**. Pathway enrichment analysis of the mRNA targets of candidate blood small EV miRNAs. **Figure S9**. Survival curves of samples with different expression levels of miR-335-5p using TCGA data. **Figure S10**. Survival curves of samples with different expression levels of miR-340-5p using TCGA data. **Figure S11**. Blood small EV miR-335-5p/miR-340-5p to distinguish between metastasis patients and non-metastasis in the test cohort.

**Additional file 2：Table S1.** Clinical features of patients in the training and test cohorts. **Table S2**. The outcome of PDAC patients in the training and test cohorts. **Table S3**. Top 15 blood small EV miRNA candidates sorted by AUC to separate PDAC from CP in training cohort. **Table S4**. The selected highly and differentially expressed miRNAs in the training cohort. **Table S5**. All pairs of candidate blood small EV miRNAs sorted by AUC in the training cohort. **Table S6**. The experimentally suppported or predicted targets of miR-95-3p and miR-26b-5p. **Table S7**. All of the candidate blood small EV miRNAs to differentiate between metastatic and non-metastatic PDAC patients in the training cohort. **Table S8**. All of the blood small EV miRNAs to distinguish between metastatic and non-metastatic PDAC patients sorted by AUC in the training cohort. **Table S9**. The P value of top 5 paired candidate miRNAs from Kaplan–Meier plot on the survival of pancreatic cancer in TCGA.
